# The utilization of the multimodal immunotherapy for the opsoclonus–myoclonus syndrome can reduce relapses and permanent neurological sequelae

**DOI:** 10.1186/s13052-025-01875-2

**Published:** 2025-02-07

**Authors:** Fang He, Miriam Kessi, Ciliu Zhang, Jing Peng, Fei Yin, Lifen Yang

**Affiliations:** 1 Department of Pediatrics, Xiangya Hospital, Central South University, Changsha, 410008 Hunan China; 2 Hunan Intellectual and Developmental Disabilities Research Center, Pediatrics, Changsha, China; 3 Clinical Research Center for Children Neurodevelopmental Disabilities of Hunan Province, Xiangya Hospital, Central South University, Changsha, China

**Keywords:** Opsoclonus–Myoclonus syndrome, Multimodal therapy, Intravenous dexamethasone, Rituximab, Intravenous immunoglobulin, Relapses, Permanent neurological sequelae

## Abstract

**Background:**

There is no known effective treatment strategy to prevent relapses and avert permanent neurological sequelae in opsoclonus–myoclonus syndrome (OMS). To describe the treatment strategy that reduced relapses and led to remission of OMS without permanent neurological sequelae.

**Methods:**

This cross-sectional study was conducted at the Department of Pediatrics, Xiangya Hospital, Central South University. Patients diagnosed with OMS from January 2014 to July 2022 were enrolled. Exposures included treatment with multimodal immunotherapy. Main outcomes and measures included the OMS severity grading scale and DQ/IQ scores. The clinical data was collected and analysed.

**Results:**

Of the six recruited patients, three were (50.00%) males. The median age of onset was 15.50 months. Preceding manifestation was present in one patient and two cases had neuroblastoma. The mean duration from disease onset to the initiation of therapies was 1.86 months. The combinations of ≥ two therapies were used: combination of the intravenous immunoglobulin (IVIG) plus intravenous dexamethasone plus rituximab was used for three (50.00%) patients and the combination of the IVIG plus intravenous dexamethasone plus rituximab plus mycophenolate mofetil for one (16.67%) case. Besides, the combination of the intravenous methylprednisolone (IVMP, which was switched to intravenous dexamethasone later) plus rituximab was utilized for one (16.67%) case and the combination of the IVIG and IVMP for one (16.67%) patient. Total numbers of the therapies used comprised of dual therapy (33.33%), triple therapy (50.00%), and other multiple agents (16.67%). Besides, surgical resections were done for the cases with tumors. The disease course was monophasic for five cases and five cases achieved remission. OMS scores improved significantly at the end of follow up. Five (83.33%) patients did not have permanent neurological sequelae.

**Conclusions:**

The combination of the intravenous dexamethasone plus IVIG plus rituximab for the OMS can reduce relapses and permanent neurological sequelae.

## Introduction

Opsoclonus–myoclonus syndrome (OMS) (also known as dancing eye syndrome or opsoclonus– myoclonus–ataxia syndrome or myoclonic encephalopathy or Kinsbourne syndrome) is a rare serious condition that can present as monophasic or chronic relapsing [1]. It has an incidence of 0.27–0.40 cases per million children [2], and the age of onset range from 1 to 3 years [3]. It is considered to be an immune-mediated disorder and can be paraneoplastic; 2–3% of the children with neuroblastoma have OMS and 50% of the OMS children have neuroblastoma [1, 4]. It tends to be acute or subacute, characterized by the rapid and chaotic eye movements, ataxia, myoclonic jerking of the limbs and trunk, vomiting, abnormal behavioral (loss of social interaction), severe irritability and sleep disturbance. It can be accompanied with abdominal or thoracic neuroblastoma or can be infectious related. Likewise, autoimmune encephalitis is an important cause of pediatric encephalopathy characterized by neuropsychiatric and neurological symptoms including movement disorders, seizures, and cognitive regression [5]. It is frequently associated with autoantibodies targeting neuronal surface or synaptic antigens [5]. A recent review showed that pediatric AE and OMS might be sharing a common immunological basis, although the former is often linked to surface antigen-targeting antibodies [5].

The mainstay treatment strategy for the OMS is immunosuppressive therapies although there is no enough clear evidence for the outcome since the condition is very rare. Immunosuppressive therapies are indicated for all OMS patients regardless of the presence or absence of the neuroblastoma. The recommended treatment strategy include the provision of the steroids (prednisolone or adrenocorticoptropic hormone (ACTH) or intravenous methylprednisolone pulses (IVMP) or oral dexamethasone pulses) within two weeks of disease onset for one year plus intravenous immunoglobulin (IVIG) and/or plasmapheresis plus rituximab or cyclophosphamide [4]. However, it worth noting that the recommended different kinds of steroids, rituximab and cyclophosphamide might have different efficacy. Besides, the mode of administration can also affect the outcome: monotherapy versus polytherapy. Consequently, there is a need to identify the optimal treatment regimen: the most effective therapies and the best mode of the administration. However, AE often responds effectively to immune therapy, underscoring the importance of early and targeted intervention [5].

Although immunosuppressive therapies and tumor resection can improve the acute OMS symptoms, there is no effective treatment to avert permanent neurological sequelae [6]. A small proportion of patients may recover fully, some can experience chronic relapses, and others can remain with minimal sequelae. Nevertheless, the majority of the patients (70–80%) remain with devastating chronic/permanent neurological disability; moderate to severe motor and cognitive difficulties and attention- deficit/hyperactive disorder which might be distressing [1, 6, 7]. Studies with 5–20 years of follow up of OMG patients showed that > 70% of the patients remained with permanent neurologic deficits including loss of speech and language, loss of coordination and abnormal eye movements. Besides, > 50% of the patients demonstrated deficits in cognition, adaptive behavior, and mild-severe intellectual disability [6]. The main challenges facing clinicians now include making an early diagnosis, providing optimal treatment early enough, reducing the chronic relapsing course and permanent neurological sequelae as well as improving the quality of life of OMS children. In this study, we have shared our experience on treatment strategy that led to remission of OMS without permanent neurological sequelae. Our study unveiled for the first time that the use of the aggressive treatment therapies; the combination of the intravenous dexamethasone pulses followed by oral prednisolone and rituximab for the OMS can prevent relapses, lead to remission and prevent the permanent neurological sequela. This study sheds more light on the potential optimal treatment that can reduce chronic relapsing course and permanent neurological sequelae and thus, improve the quality of life of OMS children.

## Methods

This retrospective study was permitted by the ethical committee of Xiangya Hospital, Central South University, and was piloted conferring the tenets of the Declaration of Helsinki. The ethical approval number 202,310,892 was obtained on 19th October 2023. Clinical data was collected from medical records. The parents/guardians of the patients provided informed written consents. All included patients were diagnosed with OMS before the age of 14 years, from January 2014 to July 2022 at the Department of Pediatrics, Xiangya Hospital of the Central South University. Patients who met the proposed diagnostic criteria for the OMS comprising the presence of three out of the subsequent four manifestations: [1] opsoclonus or ocular flutter [2], neuroblastoma [3], myoclonus and/or ataxia, and [4] behavioral and/or sleep disturbance often with marked irritability were included in this study [6]. We further included patients with at least 2 years of follow up and excluded individuals with insufficient clinical information and follow-up of < 2 years, patients with incorrect diagnosis of OMS; presented with nystagmus or unrelated eye movement abnormalities, verified seizures (not just bouts of myoclonus), acute cerebellar ataxia, or the presence of other autoimmune diseases. The data collected comprised age, sex, history of prodromal infection, history of vaccination, clinical manifestations, OMS score at onset, infection-screening results, antibodies screening results, screening for neuroblastoma results, magnetic resonance imaging (MRI) results, cerebral spinal fluid (CSF) results, blood tests results, therapies, prognosis, outcome and follow up. The OMS severity grading (Pranzatelli and Mitchell-Pike) scale with 0–18 points (assessing stance, gait, hand/arm function, opsoclonus, mood/behavior, and speech) was utilized [4].

Patients were considered to have a complete improvement when achieved total remission of symptoms; OMS score of zero and no other sequelae. Patients were considered to have a very good evolution when achieved an OMS score ≤ 2 with no other associated problems or zero but with other problems. Patients were considered to have a good evolution when achieved an OMS score ≤ 2 in linked with other neuropsychological sequalae. The term regular evolution was used for the patients whose OMS score ranged 2–6 accompanied with or without other neuropsychological problems. The term bad evolution was utilized when the patients had OMS score of > 6. The term relapse was used for the patients who scored > 0 on the OMS severity score again after prior achievement of the 0 score. The term clinical aggravation was applied for the patients with a worsening of ≥ 1 on the OMS score for more than a month [8]. Whenever possible, the development quotient (DQ) of patients was calculated by Gesell development scales and intelligence quotient (IQ) by Wechsler’s scale as per our previous studies [9, 10]. The data was processed by SPSS Version 27 software was used to summarize few data.

## Results

### Demographic and basic clinical information of the patients

We recruited six patients from our hospital: 3 (50.00%) males and 3 (50.00%) females. The median age of onset was 15.50 ± 3.0.8 SD [9–18] months. The initial clinical manifestations included opsoclonus (6, 100.00%), myoclonus (6, 100.00%), ataxia (6, 100.00%), irritability (6, 100.00%), sleep disturbance (6, 100.00%), abnormal behavior (6, 100.00%), and feeling frightened (3, 50.00%). Preceding manifestation was present in only one patient (vaccination). The OMS scores at the disease onset were 18, 18, 15, 18, 14, and 14 for the patient 1, 2, 3, 4, 5, and 6, respectively. Neuron-specific enolase was elevated for six cases (100%); four cases (66.67%) demonstrated slow background in electroencephalograph. Two cases had neuroblastoma located in the mediastinum (33.33%). There was no evidence of the pathogenic infection. Table 1 summarizes this information.

**Table 1:** Demographic and basic clinical information of the patients

Clinical feature	Patient 1	Patient 2	Patient 3	Patient 4	Patient 5	Patient 6
Age of onset	16 months	15 months	14 months	9 months	18 months	16 months
Sex	Female	Male	Female	Female	Male	Male
**Clinical manifestations**						
Opsoclonus	Yes	Yes	Yes	Yes	Yes	Yes
Myoclonus	Yes	Yes	Yes	Yes	Yes	Yes
Ataxia	Yes	Yes	Yes	Yes	Yes	Yes
Irritability	Yes	Yes	Yes	Yes	Yes	Yes
Sleep disturbance	Yes	Yes	Yes	Yes	Yes	Yes
Abnormal behavior	Yes	Yes	Yes	Yes	Yes	Yes
Feeling frightened	Yes	No	No	No	Yes	Yes
**Preceding manifestations**	None	Vaccination of varicella	None	None	None	None
**Etiology (neuroblastoma or infectious)**	Unknown	Unknown	Neuroblastoma	Unknown	Unknown	Neuroblastoma
**OMS score at presentation**	18	18	15	18	14	14
**Investigations**						
Evidence of pathogenic infection after screening	No	No	No	No	No	No
CSF analysis (antibodies)	Not available	Negative	Not available	Not available	Negative	Negative
Serum analysis (antibodies)	Negative	Negative	Not available	Negative	Negative	Negative
Neuron**-**specific enolase	Elevated	Elevated	Elevated	Elevated	Elevated	Elevated
Electroencephalograph	Normal at the beginning, slow background later.	Normal at the beginning, slow background later.	Slow background	Slow background then normal.	Normal	Normal
Targeted/whole-body MRI/CT for neuroblastoma	Normal	Normal	CT found neuroblastoma in the posterior mediastinum.	Normal	Normal	CT found neuroblastoma in the posterior mediastinum
Urine catecholamine (Vanillylmandelic acid )	Negative	Negative	Not available	Elevated	Negative	Negative

Abbreviations: CSF; cerebral spinal fluid, CT; computed tomography, MRI; magnetic resonance imaging, OMS; Opsoclonus–myoclonus syndrome

**Table 2:** Therapies and strategies used, prognosis and outcome

Clinical feature	Patient 1	Patient 2	Patient 3	Patient 4	Patient 5	Patient 6
Duration from the symptoms to treatment initiation	3 months	11 days	1 month	3 months	3 months	24 days
**Therapies used**						
Intravenous methyl prednisolone	Yes	No	Yes	No	No	No
IVIG	No	Yes	Yes	Yes	Yes	Yes
Intravenous dexamethasone	Yes	Yes	No	Yes	Yes	Yes
Rituximab	Yes	Yes	No	Yes	Yes	Yes
Mycophenolate mofetil	No	No	No	No	Yes	No
Complete surgical resection	Not applicable	Not applicable	Yes	Not applicable	Not applicable	Yes
**Sequence of the therapies used**	Intravenous methylprednisolone pulses and rituximab (1 cycle) and patient achieved remission but relapsed after 1 year and 8 months. She then received IV dexamethasone pulse and rituximab (1 cycle).	In the hospitals before coming to our hospital, he received IV dexamethasone pulse and 8 rounds of IVIG within 1 year and 9 months but the symptoms were still not controlled. At our hospital: the patient received IVIG and rituximab (1 cycle)	Tumor resection, IVIG, methylprednisolone pulses, prednisolone (low oral dose till now)	Combination of IVIG plus IV dexamethasone pulse (4 times) and rituximab for 1 cycle	Combination of IV dexamethasone pulse plus IVIG (5 times), rituximab (one cycle), then mycophenolate mofetil	IVIG, surgery, refused chemotherapy, then IV dexamethasone (pulse), rituximab (one cycle)
**Disease course (Monophasic**,** recurrent relapsing**,** chronic relapsing)**	Relapsed once	Monophasic	Monophasic	Monophasic	Monophasic	Monophasic
**Number of relapses**	1	None	None	None	None	None
**Final remission**	Remitted	Remitted	Remitted	Remitted	Remitted	Remitted
**Follow up duration**	10 years and 4 months	2 years	2 years and 8 months	6 years and 7 months	8 years and 5 months	8 years and 7 months
**Neurological sequela at last follow up**	None	None	Mild motor and mental delay	None	None	None
**OMS score at last follow up**	0	0	1	0	0	0
**DQ/IQ/school performance**	Attending normal school with normal performance	Attending normal school with normal performance	Attending normal school with low performance	Attending normal school with normal performance	Attending normal school with normal performance	Attending normal school with normal performance

Abbreviations: IV; intravenous, IVIG; intravenous immunoglobulin, OMS; opsoclonus–myoclonus syndrome

### Therapies used, outcome and prognosis

**Fig. 1:**
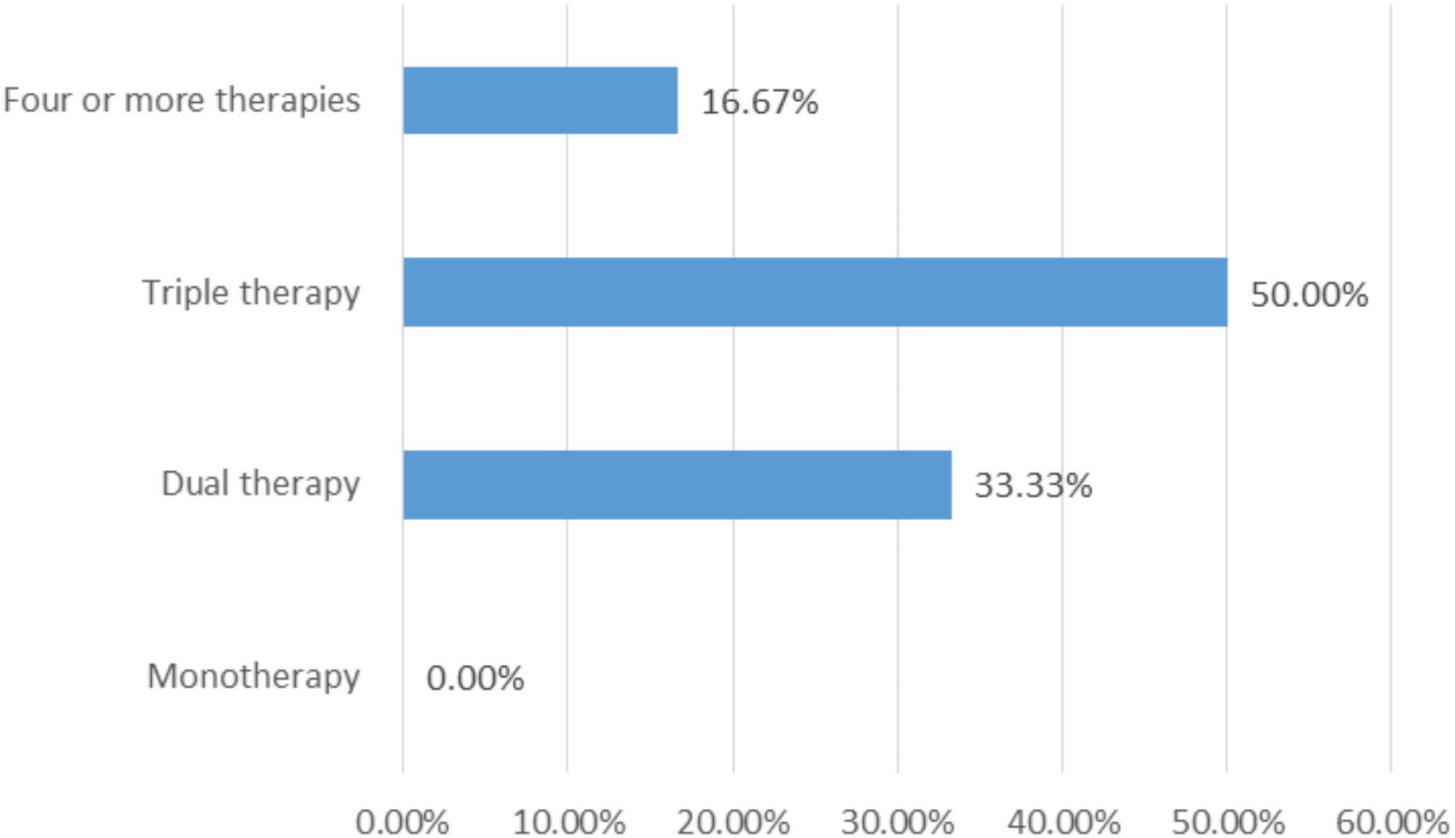
The categories of the treatment used

**Figure 2:**
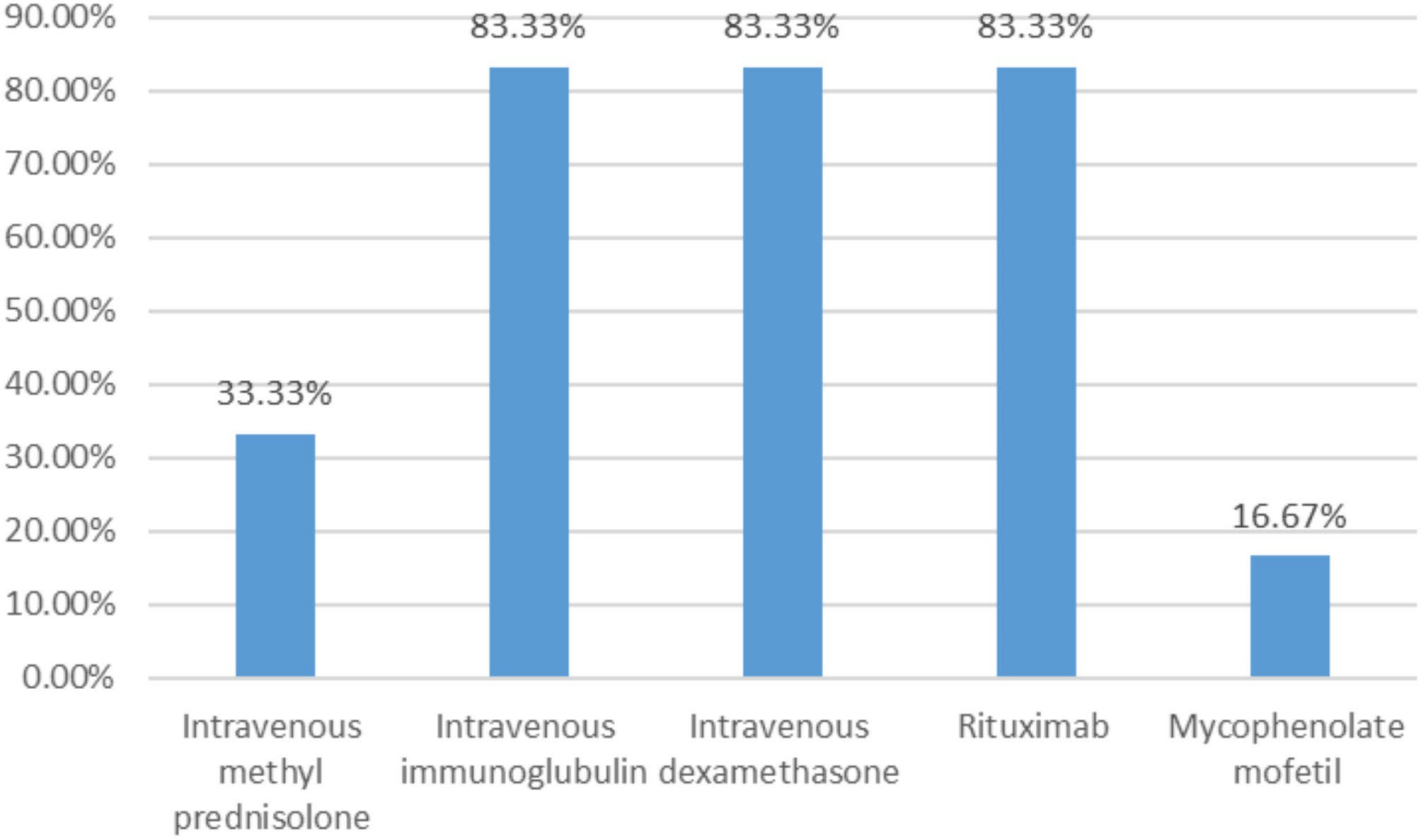
Frequency of therapies used

The mean duration from disease onset to the initiation of therapies was 1.86 ± 0.1.26 SD (range 0.37-3.00) months. The combinations of ≥ two therapies were used at our hospital and were repeated at least monthly until the symptoms were controlled. The combination of the IVIG plus intravenous dexamethasone plus rituximab was used for three (50.00%) patients and the combination of the IVIG plus intravenous dexamethasone plus rituximab plus mycophenolate mofetil for one (16.67%) case. In addition, the combination of the IVMP which was then switched to intravenous dexamethasone later plus rituximab for one (16.67%) case and the combination of the IVIG and IVMP for one (16.67%) patient. Total numbers of the therapies used at last follow up comprised monotherapy (0, 0.00%), dual therapy (2/6, 33.33%), triple therapy (3/6, 50.00%), and other multiple agents (1/6, 16.67%) (Fig. 1). Therapies used included IVMP (2/6, 33.33%), IVIG (5/6, 83.33%), intravenous dexamethasone (5/6, 83.33%), rituximab (5/6, 83.33%) and mycophenolate mofetil (1/6, 16.67%) (Fig. 2). Besides, complete surgical resections were done for the cases with tumors (2, 100.00%). Some patients required many cycles of treatment while others did not. The disease course was monophasic for five cases and five cases achieved remission. OMS scores at last follow up were 0, 0, 1, 0, 0, and 0 for the patient 1, 2, 3, 4, 5, and 6, respectively. Five (83.33%) patients did not have permanent neurological sequela; they attended normal school with normal performance. Besides, only patient 3 remained with mild motor and mental delay at last follow up (Table 2).

## Discussion

This study included six cases; male to female ratio (1:1) and the median age of onset was 15.50 months. The initial clinical manifestations included opsoclonus (100.00%), myoclonus (100.00%), ataxia (100.00%), irritability (100.00%), sleep disturbance (100.00%), abnormal behavior (100.00%), and frightened feeling (50.00%). Two cases had neuroblastoma located in the mediastinum (33.33%) while the rest were probably idiopathic. The mean duration from disease onset to the initiation of therapies was 1.86 months. The combinations of ≥ two therapies were used at once and were repeated at least monthly until the symptoms were controlled: IVIG plus intravenous dexamethasone plus rituximab (50.00%). At last follow up, dual therapy was used for two cases (33.33%), triple therapy for 3 cases (50.00%) and one case (16.67%) received multiple agents (≥ 4 therapies). Therapies used included intravenous IVMP (33.33%), IVIG (83.33%), intravenous dexamethasone (83.33%), rituximab (83.33%), mycophenolate mofetil (16.67%) and complete surgical resection (100.00%). The disease course was monophasic for five cases and five cases achieved remission. Five (83.33%) patients did not have permanent neurological sequela and only one patient remained with mild motor and mental delay at last follow up. To the best of our knowledge, this is the first study to show that the use of the aggressive treatment therapies (the combination of the intravenous dexamethasone plus IVIG plus rituximab) for the OMS can eliminate/reduce the permanent neurological sequela.

The male to female ratio was 1:1 in our study but there are some conflicting findings from the previous reports. It has been reported that there is both female [2, 11–13] and male predominance [8, 14]. The median onset age of our patients was 15.50 months while it ranged from 16 to 20 months based on other studies [2, 8, 11–14]. Similar to other previous studies [2, 11, 12], our patients presented with cardinal OMS symptoms with the exception of the frightened feeling. Notably, although ataxia is not a common neurological emergency in pediatric population, it is important to make an early diagnosis and provide proper management for the serious ataxia-related conditions including OMS. A recent multicenter study involving 509 patients aged 1–18 years conducted in Italy with the aim of investigating causes of acute ataxia unveiled that, OMS is among the causes: it contributes approximately 7.5% of all causes [15]. Tumor detection rate was 33.33% in our study, 50.00% in another US study (*N* = 358) [11], 44.44% in another Chinese study (*N* = 9) [12], 43.5% in Japanese study (*N* = 23) [2], and 45% in Spanish study [13] suggesting the relationship between tumors and OMS.

The disease course was monophasic for 83.33% of our patients. The combinations of ≥ two therapies were used in our study and five cases (83.33%) achieved remission without permanent neurological sequela. In another US study that included 358 cases, 48.00% received monotherapy, 37.00% dual therapy, 11.00% triple therapy and 8.00% other multiple agents and secondary outcome categories included 28.00% of cases had mild score, 41.00% had moderate score and 31.00% has severe score [11]. Therapies used in their study included corticosteroids only (15.00%), corticotropin only (18.00%), IVIG only (15.00%), steroids and IVIG only (17.00%), corticotropin and IVIG only (20.00%), rituximab (28.00%), cyclophosphamide (57.00%), azathioprine (6.00%) and mycophenolate mofetil (9.00%) [11]. Similar to our study, 83.33% (10/12) of the OMS patients that received multimodal immunotherapy (steroids, IVIG and azathioprine or cyclophosphamide or rituximab had no/minimal neurological sequelae [16]. In another study where only 4 of the 22 cases with neuroblastoma used multiple therapies: corticotherapy (*n* = 33), IVIG (*n* = 13), cyclophosphamide (*n* = 4) and rituximab (*n* = 4)), 59.00% of the cases had neurological sequelae [17]. In another study that included 14 cases of whom 12 received the combination of corticotropin plus IVIG and the combination of three oral steroids plus IVIG revealed that the use of more aggressive immunosuppression caused patients to function at or near normal [18].

In this study, patient 3 did not receive both intravenous dexamethasone and rituximab and ended up with mild motor and mental delay which corresponds with findings from other studies. In a Chinese study that included 9 cases, the initial combination of the IVIG and IVMP improved symptoms but they had several relapses during steroids tapering process, however, the administration of the rituximab resulted to relapse free to all four (100.00%) cases; improved recognition and long-term outcome [12]. Nevertheless, all nine cases in the same study remained with at least one neurological symptoms [12] probably due to lack or delayed introduction of rituximab. In another case series of 14 cases with neuroblastoma of whom 10 received immunotherapies: (4/10) IVMP, (6/10) prednisone, (1/10) adrenocorticotropic hormone, IVIG (8/10) and (1/10) rituximab, symptoms improved in all patients [14]. Nevertheless, relapses were observed during immunotherapy weaning and (93.00% remained with neurological sequelae may be because of lack of rituximab use for the majority of the cases [14]. In another France study involving 13 cases, the distribution of therapies included corticosteroids (*n* = 13), tumor resection (*n* = 5) and immunotherapy (*n* = 3), as a results only 38.46% achieved full recovery while 38.46% remained with neurodevelopmental sequelae [8]. In a Japanese study that included 23 patients, 73.9% used IVIG whereby 35.30% achieved complete remission, 56.5% used IVMP of whom 23.1% achieved complete remission, 52.2% received oral prednisolone of whom 33.3% achieved complete remission, 26.1% received chemotherapy and/or tumors resection of whom 66.7% achieved complete remission and 8.7% used rituximab of whom 100% achieved complete remission [2]. However, at the end of follow up 70% had neurological sequelae [2] probably because of lack of use of multimodal therapy, lack of use of rituximab for some cases and delayed treatment initiation. In Poland study that involved 7 cases, the combination of cyclophosphamide plus dexamethasone resulted to a complete remission in 4 children while 3 cases remained with neurological sequelae [19] suggesting that rituximab might be superior to cyclophosphamide. It worth noting that, intravenous dexamethasone and rituximab are sometimes not adequate for severe cases as observed in our patient 4 and 5, consequently, the addition of IVIG is inevitable. Altogether, our study and previous studies suggest the use of the multimodal therapy particularly the combination of intravenous dexamethasone plus IVIG plus rituximab.

In comparison to other studies, the good outcome in our study might be attributed by an early initiation of therapies, which reduced relapse rate (only one of our patients relapsed) as well as lack of use of the adrenocorticotropic hormone. In this study, the mean duration from disease onset to the initiation of therapies was 1.86 months, whereas, it was 16 months in another study [11] and 14 days in another study [8]. The commencement of the treatment at > 30 weeks has been related to severe neurological sequelae according to the Japanese study [2]. It has been shown that multiple relapses (> 3) and late tumor resection (> 6 months after symptom onset) are related to poor outcome [20]. The high recurrence/relapse rates and chronic neurologic deficits have been linked with the use of the adrenocorticotropic hormone in 91.00% of the cases [21, 22] although it is currently among the recommended therapies as per guideline [4]. Besides, IVMP has been reported to be less effective for OMS than pulse dexamethasone [8], which corresponds to the patient 1 who experienced one relapse possibly because she did not receive both pulse dexamethasone and rituximab early enough. Three of our patients (patient 4, 5 and 6) received the combination of intravenous dexamethasone plus IVIG plus rituximab at the very beginning and achieved full remission within one year without relapse and neurological sequelae. It has been shown that number of relapses are negatively linked with IQ in children [23]. Although we employed multimodal therapy in our study, none of our patients experienced severe side effects because the main therapies were administered within a short period of time (completion of therapies within one year). Consequently, the ultimate goal of OMS treatment is elimination of relapse, which can be achieved by administration of the multimodal therapy suggested above (combination of pulse dexamethasone plus IVIG plus rituximab). Barbagallo M et al. demonstrated that pediatric AE and OMS might have an overlapping immunological basis, therefore, aggressive therapeutic approach employed for OMS in this study, including multimodal strategies with rituximab, may also be applied in managing other neuroimmunological conditions, improving clinical outcomes [5]. Since OMS is among the causes of acute ataxia in children [15], the multimodal treatment strategies utilized in this study can be employed to the patients early enough to prevent some long-term complications.

## Conclusions

The use of the aggressive treatment therapies; the combination of the intravenous dexamethasone plus IVIG plus rituximab for the OMS can reduce relapses and permanent neurological sequelae. In light of the similarities with other autoimmune encephalopathies, as discussed by Barbagallo et al. [5], our study reinforces the importance of early recognition of pediatric neuroimmunological disorders and initiating multimodal immunotherapy to mitigate permanent neurological sequelae.

### Study limitations

The study was conducted retrospectively; therefore, it is prone to information bias. It involved a small sample size; however, due to rarity of the OMS and main challenges facing clinicians now including the lack of knowledge of optimal treatment for reducing chronic relapsing course and permanent neurological sequelae, the study design and the small sample size was inevitable. Prospective multicenter studies are needed to assist more in the guidance of the treatment of the OMS cases.

## Data Availability

All data generated or analysed during this study are included in this published article.

## Abbreviations

OMS: Opsoclonus–myoclonus syndrome
IVIG: Intravenous immunoglobulin
IVMP: Intravenous methylprednisolone
ACTH: Adrenocorticoptropic hormone
MRI: Magnetic resonance imaging
CSF: Cerebral spinal fluid
